# P44, the ‘longevity-assurance’ isoform of P53, regulates tau phosphorylation and is activated in an age-dependent fashion

**DOI:** 10.1111/acel.12192

**Published:** 2014-02-25

**Authors:** Mariana Pehar, Mi Hee Ko, Mi Li, Heidi Scrable, Luigi Puglielli

**Affiliations:** 1 Department of Medicine, University of Wisconsin-Madison2500 Overlook Terrace, Madison, WI, 53705, USA; 2 Robert and Arlene Kogod Center on Aging, Division of Experimental Pathology, Mayo ClinicRochester, MN, 55905, USA; 3 Geriatric Research Education Clinical Center, VA Medical Center2500 Overlook Terrace, Madison, WI, 53705, USA; † Department of Cell and Molecular Pharmacology and Experimental Therapeutics, Medical University of South CarolinaCharleston, SC, 29425, USA; ‡ Division of Bioresources Research, Jeju Technopark, Jeju Biodiversity Research Institute338 Sinryedong-ro Namwon-eup, Seogwipo-si, Jeju-do, 699-943, Korea

**Keywords:** aging, Alzheimer’s disease, cognitive decline, p44, p53, tau

## Abstract

p44 is a short isoform of p53 with ‘longevity-assurance’ activity. Overexpression of p44 in the mouse (p44^+/+^ transgenic mice) causes a progeroid phenotype that mimics an accelerated form of aging. The phenotype includes abnormal phosphorylation of the microtubule-binding protein tau, synaptic deficits, and cognitive decline. Genetic engineering demonstrated that the phosphorylation status of tau acts upstream of the synaptic deficits. Here, we provide evidence that p44 promotes the phosphorylation of tau in the mouse. Specifically, we show that p44 binds to the promoter of tau kinases Dyrk1A, GSK3β, Cdk5, p35, and p39 and activates their transcription. The upregulation of the above kinases is followed by increased phosphorylation of tau. Finally, we show that p44 is preferentially found in the nucleus and that its levels increase with age in the mouse brain. Taken together, these results suggest that an imbalance in the p53:p44 ratio might be involved with the altered tau metabolism that characterizes aging.

## Introduction

The p53 protein is best known for its tumor suppressor activity. In fact, a large number of mutations associated with human cancers are found in the p53 gene (*TP53*). However, recent findings have revealed that *TP53* undergoes a combination of alternative promoter usage and alternative initiation of translation resulting in four major isoforms, full-length p53 (referred to as p53 thereafter), ∆40p53, ∆133p53, and ∆160p53. ∆40p53, ∆133p53, and ∆160p53 are N-terminally truncated versions of full-length p53 lacking the first 39, 132, and 159 amino acids, respectively (Bourdon, 2007; Marcel *et al*., 2010). As a result of the above truncations, ∆40p53 lacks the first transactivation domain (TD1) of p53 but retains the second (TD2); in contrast, ∆133p53 and ∆160p53 lack both transactivation domains but retain almost entirely the DNA-binding region.

∆40p53 (referred to as p44 thereafter) has received increased attention because of its role in aging and Alzheimer’s disease (AD) (Maier *et al*., 2004; Costantini *et al*., 2006; Pehar *et al*., 2010). In fact, overexpression of p44 in the mouse (p44^+/+^ transgenic mice) causes a progeroid phenotype that resembles an accelerated form of aging (Maier *et al*., 2004). This phenotype is reversed when p44^+/+^ mice are crossed with mice lacking endogenous *TP53* (p53^−/−^ mice), indicating that p44 requires full-length p53 to exert its ‘proaging’ actions (Maier *et al*., 2004). The fact that several other mouse models with altered p53 activity develop a similar progeroid phenotype supports the conclusion that the tumor suppressor gene *TP53* has also longevity-assurance activity (Bauer & Helfand, 2006; Rodier *et al*., 2007; Ungewitter & Scrable, 2008). In addition to the accelerated aging and reduced lifespan, p44^+/+^ mice display hyperphosphorylation of the microtubule-binding protein tau, synaptic impairment, and premature cognitive decline (Pehar *et al*., 2010). When engineered to express a humanized version of mouse amyloid precursor protein (APP), a protein that is tightly linked to AD pathogenesis, p44^+/+^ mice develop a severe form of neurodegeneration affecting memory-forming and memory-retrieving areas of the brain (Pehar *et al*., 2010).

It is currently unclear how increased levels of p44 in the brain may lead to synaptic defects. However, the fact that they can be rescued by the haploinsufficiency of *Mapt* (the gene encoding tau) suggests a possible mechanistic connection between p44 and tau (Pehar *et al*., 2010). It is also worth stressing that *Mapt* mutations have been associated with hereditary forms of frontotemporal dementias and that tau polymorphisms appear to act as genetic risk factors for sporadic progressive supranuclear palsy and corticobasal degeneration (Lee *et al*., 2001). Abnormal phosphorylation and aggregation of tau in the form of neurofibrillary tangles (NFT) is also an essential feature of AD [reviewed in (Pehar & Puglielli, 2012)]. Finally, abnormal tau processing has been linked to the memory deficits of different mouse models of human neurodegenerative diseases (Lee *et al*., 2001; Kurz & Perneczky, 2009).

In conclusion, elucidation of the molecular mechanisms responsible for the abnormal phosphorylation of tau in p44^+/+^ mice would likely yield crucial and novel aspects of tau regulation and its role in age-related cognitive impairment as well as different forms of neurodegenerative diseases.

## Results

**Figure 1:**
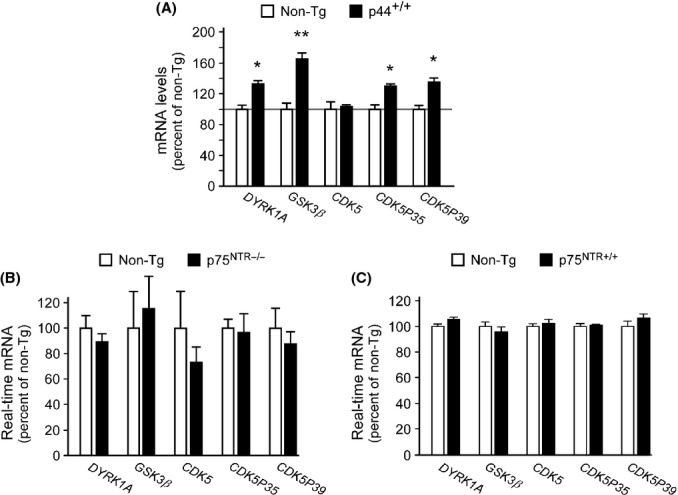
Transgenic mice overexpressing p44 (p44^+/+^) display increased mRNA levels of *DYRK1A*, *GSK3*β, *CDK5P35,* and *CDK5P39*. Quantitative real-time PCR determination of indicated kinases in the hippocampal formation. (A) Nontransgenic (non-Tg) and p44^+/+^ mice. (B) Non-Tg and p75^NTR−/−^ mice. (C) Non-Tg and p75^NTR+/+^ mice. Animals were 2.5 months old when analyzed. All values are mean (*n* = 5) ± SD. *P < 0.05; **P < 0.005.

To acquire more insight on the mechanisms responsible for the abnormal phosphorylation of tau in p44^+/+^ mice, we analyzed the mRNA levels of dual-specificity tyrosine-regulated kinase 1a (Dyrk1a), glycogen synthase kinase-3β (GSK3β), and cyclin-dependent kinase 5 (Cdk5). Although many kinases can phosphorylate tau proteins *in vitro,* we limited our analysis to Dyrk1a, Cdk5, and GSK3β because they can phosphorylate tau *in vivo* (in the mouse brain) and are altered in late-onset AD brains (Lee *et al*., 2001; Ferrer *et al*., 2005; Schmid *et al*., 2006; Ballatore *et al*., 2007; Ryoo *et al*., 2007; Hooper *et al*., 2008). Real-time assessment of the mRNA levels of the above kinases showed that both *DYRK1A* and *GSK3*β are upregulated in the hippocampus of p44^+/+^ animals (Fig. 1A). Although *CDK5* itself did not appear to be upregulated, we observed increased mRNA levels of *CDK5P35* and *CDK5P39* (Fig. 1A), which are important regulatory partners of Cdk5 (Schmid *et al*., 2006). The magnitude of changes detected is consistent with the fact that the abnormal tau phosphorylation described in p44^+/+^ mice was limited to neurons (Pehar *et al*., 2010), which account for only 2–15% of the total mass of the brain (Azevedo *et al*., 2009).

We previously reported that p44^+/+^ animals overproduce the amyloid β-peptide (Aβ) as a result of increased expression of the neurotrophin receptor p75^NTR^ (Costantini *et al*., 2006). As p75^NTR^ signaling has also been implicated with tau hyperphosphorylation in isolated hippocampal neurons (Saez *et al*., 2006), we assessed whether the levels of the above kinases changed in relation to the expression of p75^NTR^ itself. However, the mRNA levels of the above kinases were not affected by either the genetic disruption (p75^NTR−/−^) or overexpression (p75^NTR+/+^) of p75^NTR^ in the mouse (Fig. 1B and C), suggesting that p75^NTR^ is not responsible for the transcriptional activation of Dyrk1a, GSK3β, p35, and p39 observed in p44^+/+^ animals.

**Figure 2:**
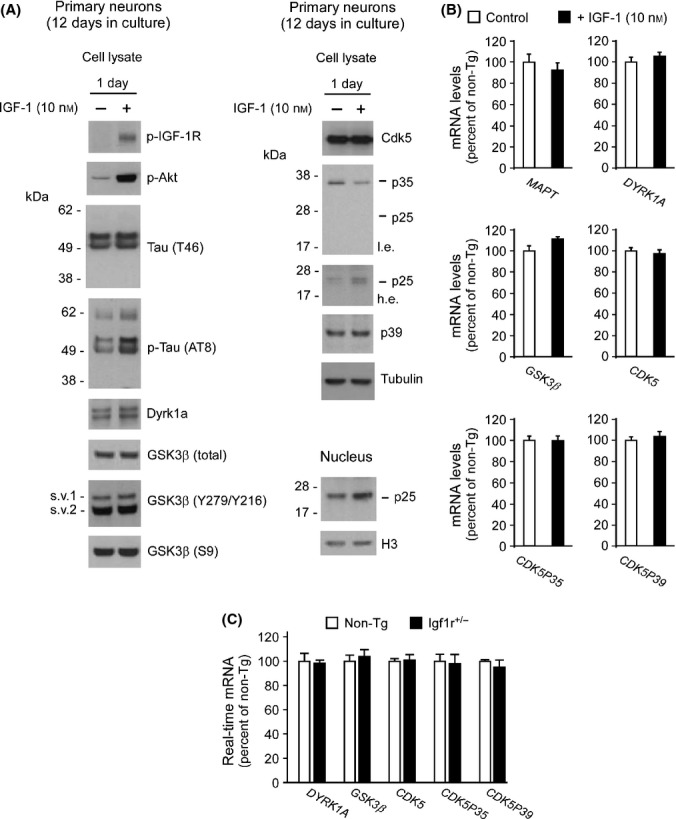
The upregulation of tau kinases in p44^+/+^ mice is independent of IGF-1R. (A, B) Primary cortical neurons from wild-type/nontransgenic mice were cultured *in vitro* and treated with IGF-1 for 1 day prior to Western blot (A) or real-time PCR (B). (C) mRNA levels of the indicated kinases in the hippocampal formation of non-Tg and Igf1r^+/−^ mice. Animals were 2.5 months old when analyzed. All values are mean ± SD. *l.e*., low exposure; *h.e*., high exposure.

The premature aging and reduced lifespan of p44^+/+^ animals have been linked – at least in part – to the hyperactivation of the insulin-like growth factor 1 receptor (IGF-1R) signaling pathway (Maier *et al*., 2004). In addition, p44^+/+^ mice display increased levels and activity of IGF-1R in the hippocampal formation (Pehar *et al*., 2010). Therefore, it is possible that the abnormal phosphorylation of tau observed in p44^+/+^ mice is due to altered IGF-1R signaling. To assess this possibility, we treated cultured hippocampal neurons with 10 nm IGF-1 and determined both activation of IGF-1R signaling and levels of the above kinases. Figure 2A shows that activation of IGF-1R signaling resulted in increased processing of p35 into the shorter p25 (see low and high exposures of Fig. 2A). Because p25 preferentially distributes in the nucleus, we confirmed the increased generation of p25 by assessing its protein levels in the nuclear fraction of IGF-1-treated neurons (Fig. 2A). An increased conversion of p35 into p25 has been linked to the activation of Cdk5 and hyperphosphorylation of tau in both *ex vivo* and *in vivo* systems (Schmid *et al*., 2006), and has been implicated in AD neuropathology (Patrick *et al*., 1999). Consistently, we found increased phosphorylation of tau following treatment with IGF-1 (Fig. 2A; see p-Tau). However, we did not observe changes in the protein levels of the other kinases (Fig. 2A) and more importantly, we did not observe changes in their mRNA levels (Fig. 2B). Therefore, we conclude that although IGF-1R signaling can affect the phosphorylation status of tau, perhaps through p25-mediated activation of Cdk5 and/or other non-defined mechanisms, the increased transcription of the *DYRK1A*, *GSK3*β, *CDK5P35*, and *CDK5P39* genes observed in p44^+/+^ mice was not linked to the hyperactivation of IGF-1R. To further confirm this conclusion we also analyzed the mRNA levels of the above tau kinases in mice lacking one copy of *Igf1r* and found no significant changes (Fig. 2C).

When taken together, the above data suggest that neither p75^NTR^ nor IGF-1R is involved in the p44-dependent upregulation of the tau kinases assessed in this study. Therefore, we decided to investigate whether p44 itself could act as a transcriptional activator, either alone or by interacting with full-length p53. On this regard, it is worth noting that the mechanisms underlying p44 activity are still largely unknown. Although some of them might require full-length p53 (Maier *et al*., 2004), others might not (Ohki *et al*., 2007). A similar issue exists when considering its transcriptional activity. In fact, even though p44 retains the second transactivation domain and the DNA-binding domain of the full-length protein, it is unclear whether it has transcriptional activity and, if it does, whether it is p53-dependent or p53-independent.

**Figure 3:**
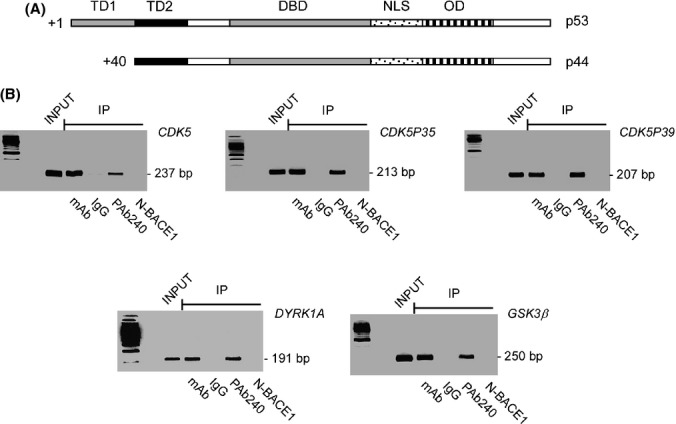
ChIP analysis – p44 and/or p53 can bind to the promoters of *CDK5*, *CDK5P35, CDK5P39, DYRK1A,* and *GSK3*β. (A) Schematic view of p53 and p44 proteins. *TD1*, transactivation domain 1; *TD2*, transactivation domain 2; *DBD*, DNA-binding domain; *NLS*, nuclear localization signal; *OD*, oligomerization domain. (B) ChIP analysis was performed with two different antibodies targeting p53/p44 (mAb and PAb240). The PCR was performed on DNA fragments isolated both before (INPUT) and after immunoprecipitation (IP). As negative controls, the IP was also performed using mouse IgG and an antibody against the N-terminal domain of the β-secretase BACE1 (N-BACE1).

To assess whether p44 physically associates with the promoter region of the above tau kinases *in vivo*, we initially performed a chromatin immunoprecipitation (ChIP) assay. Protein-DNA complexes were immunoprecipitated with two monoclonal antibodies: one raised against recombinant p53 (mAb) and the other raised against a peptide corresponding to the DNA-binding domain of p53 (PAb240). A schematic view of the domain organization of p53 and p44 is shown in Fig. 3A. DNA was extracted from semipurified complexes and amplified with specific primers designed to overlap the promoter region of *DYRK1A*, *GSK3*β, *CDK5*, *CDK5P35*, and *CDK5P39*. Figure 3B shows that we were able to generate a PCR product of the expected size with all kinases studied here. Importantly, we did not generate any product when the immunoprecipitation was performed with IgG or an antibody against a negative control that lacks transcriptional activity (the N-terminal ectodomain of BACE1; Fig. 3B). Although these results indicate that a p53 family member can bind to the promoter region of the above kinases, they cannot discriminate between the different isoforms, more specifically between p53 and p44. In fact, the antibodies used to immunoprecipitate the protein:DNA complexes are not isoform-specific (see Fig. 3A).

**Figure 4:**
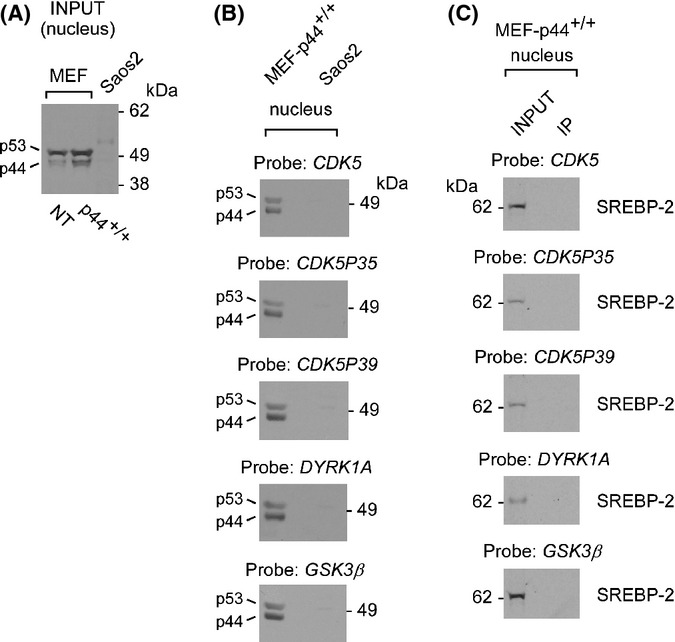
DNA:protein pull down – both p44 and p53 can bind to the promoters of *CDK5*, *CDK5P35, CDK5P39, DYRK1A,* and *GSK3*β. (A) Western blot showing the migration profile of p53 and p44 on gel electrophoresis. Neither protein was detected in p53-null Saos2 cells. (B, C) Western blot of DNA:protein pull down performed with the indicated probes. Internal controls included p53-null cells Saos2 (B) and SREBP-2 (C).

To confirm the results obtained with the ChIP assay, we conducted the reciprocal experiment. DNA probes specific to the promoter regions of the above kinases were biotinylated prior to incubation with a nuclear extract of mouse embryonic fibroblasts (MEFs) from p44^+/+^ mice. The biotinylated DNA:protein complex was then purified with streptavidin, digested with DNAses, and analyzed by SDS–PAGE and immunoblotting with antibody PAb240, which recognizes the DNA-binding domain of p53 (see Fig. 3A). We used MEF-p44^+/+^ to improve the detection of p44. Saos2 cells, which do not express p53 proteins (Masuda *et al*., 1987), were used as control. Figure 4A shows the migration of both isoforms on electrophoresis. As expected (Maier *et al*., 2004), MEF-p44^+/+^ also displayed slightly higher levels of p53. In fact, p44 has increased half-life and is known to stabilize p53 and increase its steady-state levels (Ungewitter & Scrable, 2008). As expected, none of the p53 isoforms were detected in Saos2 cells.

The DNA:protein pull down was successful with all kinases studied here (Fig. 4B). We detected both p53 and p44 isoforms indicating that both proteins are able to interact with the promoter region of target genes (*DYRK1A*, *GSK3*β, *CDK5*, *CDK5P35*, and *CDK5P39*; Fig. 4B). We never detected ∆133p53 or ∆160p53 isoforms (data not shown) indicating that only p53 and p44 can interact with the promoter region of the above genes. Neither p53 nor p44 were pulled-down in p53-null Saos2 cells (Fig. 4B). As additional control, we also attempted to pull-down sterol regulatory element-binding protein-2 (SREBP-2), a transcription factor that regulates both biosynthesis and uptake of cholesterol (Goldstein *et al*., 2006). Although we were able to detect SREBP-2 in the nuclear extract (Fig. 4C; INPUT), we were not able to pull it down with the probes employed here (Fig. 4C), supporting the validity and specificity of the results obtained with p53 and p44.

**Figure 5:**
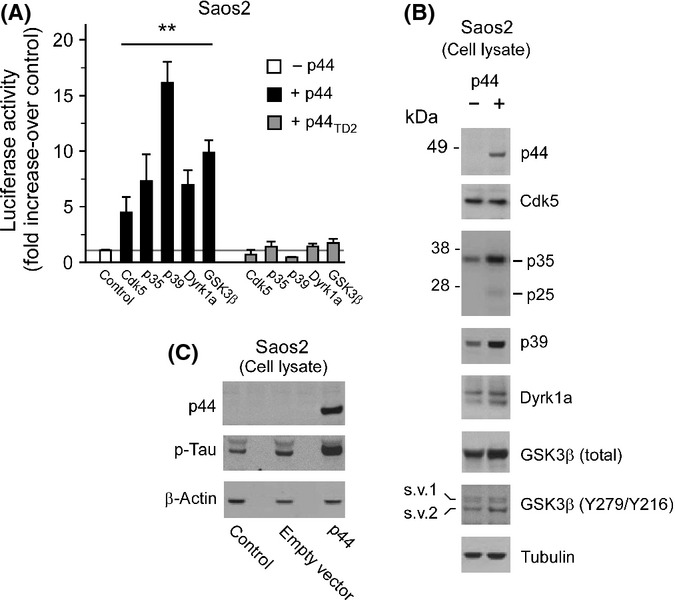
Luciferase reporter assay – p44 can activate transcription of *CDK5*, *CDK5P35, CDK5P39, DYRK1A,* and *GSK3*β. (A) The luciferase activity of the indicated promoter constructs was measured after transfection of p44 into p53-null Saos2 cells and normalized to *Renilla* luciferase activity (for transfection efficiency). A mutant version of p44 lacking TD2 (p44_TD__2_) was used as control. Values are mean ± SD. **P < 0.005 (vs. control). (B, C) Western blot analysis of p53-null cells Saos2 48 h after transfection with p44.

When taken together, the above results with ChIP and DNA:protein pull down indicate that both p53 and p44 can bind to the promoter region of *DYRK1A*, *GSK3*β, *CDK5*, *CDK5P35*, and *CDK5P39*. However, it is still possible that p44 is devoid of intrinsic transcriptional activity and that the activation of the above kinases *in vivo* is caused by the p53:p44 complex rather than p44 itself. To address this issue, we performed a dual luciferase activity assay in Saos2 cells, which lack endogenous p53 ((Masuda *et al*., 1987); see also Fig. 4A) and as a result, are ideal to differentiate between p53-dependent and p53-independent events. The promoter region of *DYRK1A*, *GSK3*β, *CDK5*, *CDK5P35*, and *CDK5P39* used for both the ChIP and DNA:protein pull down experiments was subcloned into the promoterless fire fly luciferase reporter vector pGL3 basic. The luciferase activity of the different constructs was then measured after transfection. The results shown in Fig. 5A indicate that transfection of p44 was able to increase the luciferase activity of the different targeted promoters supporting the conclusion that p44 can activate the above kinases independently of p53. No effect was observed when we used a deleted version of p44 lacking the transactivation domain (Fig. 5A).

To further confirm our results, we transfected p44 into p53-null Saos2 cells and assessed protein levels of the above tau kinases. Overexpression of p44 was accompanied by the upregulation of p35, p39, Dyrk1A, and GSK3β. No apparent effect was observed with Cdk5 (Fig. 5B). Finally, overexpression of p44 was also accompanied by increased phosphorylation of tau (Fig. 5C). Again, the fact that these results were observed in p53-null cells (Saos2) indicates that p44 can regulate the expression levels of tau kinases as well as the phosphorylation status of tau independently of full-length p53.

When taken together, the above data (obtained with ChIP, DNA:protein pull down and luciferase assays) support the conclusion that the transcriptional activation of *DYRK1A*, *GSK3*β, *CDK5P35,* and *CDK5P39* observed in p44^+/+^ mice is directly dependent on the transcriptional activity of p44 and/or the p53:p44 transcriptional complex. The fact that *CDK5* was not upregulated in the mouse brain (Fig. 1A) or in p53-null cells (Fig. 5B) might be explained by the presence of additional transcriptional or translational regulators that maintain the levels of Cdk5 under strict control.

**Figure 6:**
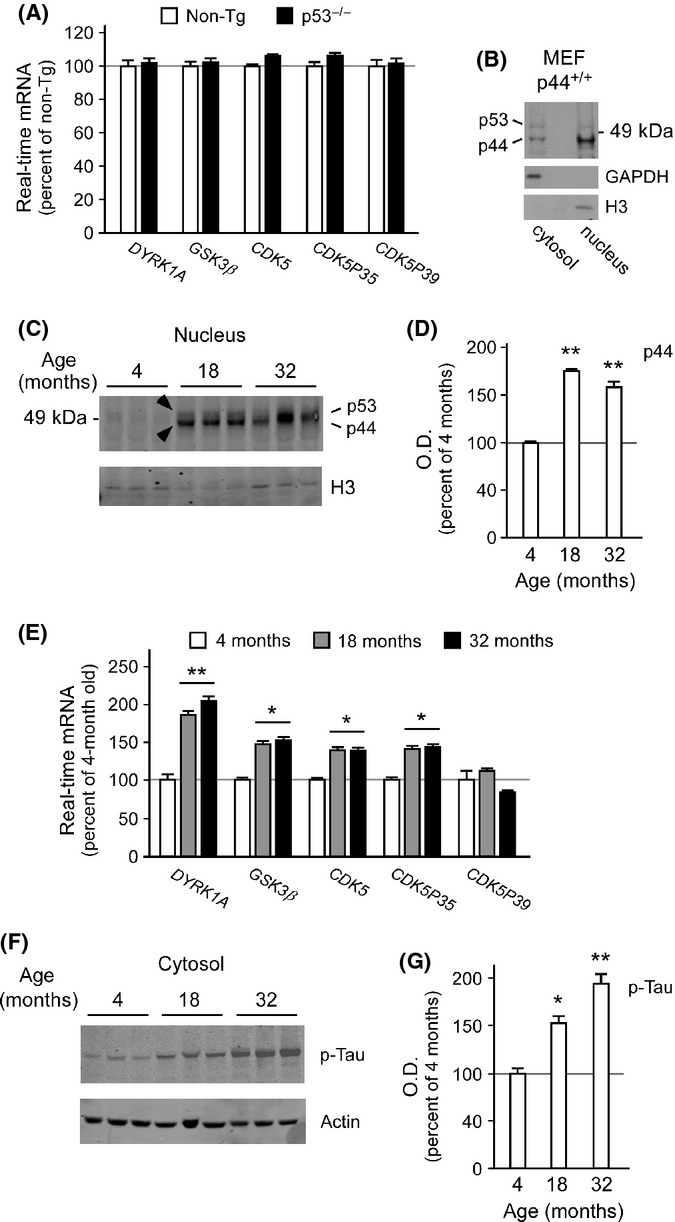
p44 levels in the mouse brain increase as a function of age. (A) Baseline mRNA levels of the indicated kinases in the brain of nontransgenic (Non-Tg) and p53^−/−^ mice. Values are mean ± SD. (B) Western blot showing preferential distribution of p44 in the nucleus. (C, D) Western blot showing nuclear levels of p53 and p44 in the brain of wild-type/non-Tg mice. Representative Western blots are shown in (C) while quantification is shown in (D). Values in (D) are mean (*n* = 3) ± SD and were normalized for H3 (nuclear marker). (E) mRNA levels of indicated kinases in the brain of wild-type/non-Tg mice. (F, G) Western blot showing cytosolic levels of p-Tau in the brain of wild-type/non-Tg mice. Representative Western blots are shown in (F) while quantification is shown in (G). Values in (G) are mean (*n* = 3) ± SD and were normalized for actin (cytosolic marker). *P < 0.05; **P < 0.005.

Although p44 and/or the p53:p44 can induce the expression of the above tau kinases (through a transcriptional mechanism), it is unclear whether they also control their baseline levels. To assess this issue, we analyzed the mRNA levels of *DYRK1A*, *GSK3*β, *CDK5*, *CDK5P35*, and *CDK5P39* in the hippocampus of p53^−/−^ mice. However, we did not detect significant changes (Fig. 6A). These results seem to oppose those generated by overexpressing p44 in the mouse (Fig. 1A) and in p53-null cells (Fig. 5) where we did observe upregulation of Dyrk1A, GSK3b, p35, and p39. Therefore, we conclude that neither p53 nor p44 is necessary to maintain the baseline levels of the kinases.

Finally, we investigated whether p44 is activated in an age-dependent manner. In fact, the phosphorylation status of tau increases with aging in the normal brain as well as in AD brains. It is also worth remembering that aging is the most important risk factor for late-onset AD (Pehar & Puglielli, 2012). As it is already known that the different p53 isoforms distribute differently between the cytosol and the nucleus (Bourdon *et al*., 2005), we first assessed whether p44 is preferentially found in the cytosol or in the nucleus. To improve detection of p44, we performed these initial experiments in cultured MEFs from p44^+/+^ mice. The results clearly indicate a preferential localization of p44 in the nuclear fraction (Fig. 6B). Next, we assessed levels of p53 and p44 in nuclear extracts from the frontal cortex of wild-type/nontransgenic mice. The results show a significant upregulation of p44 as a result of normal aging (Fig. 6C,D) that was accompanied by a parallel increase in the mRNA levels of tau kinases (Fig. 6E) as well as in phospho-tau (Fig. 6F,G). When taken together, the above findings suggest that an imbalance in the p53:p44 ratio might be involved with the altered tau metabolism that characterizes aging.

## Discussion

p44 is a short isoform of the tumor-suppressor and longevity-assurance gene *TP53* that on gel electrophoresis migrates with an apparent molecular mass of 44-kDa (mouse) or 47-kDa (human). It lacks the first transactivation domain but retains the second (Bourdon, 2007). Overexpression of p44 in the mouse results in a progeroid animal model (p44^+/+^ transgenic mice) that mimics an accelerated form of aging (Maier *et al*., 2004). The phenotype includes abnormal phosphorylation of the microtubule-binding protein tau, premature synaptic deficits, cognitive decline, and propensity to AD-like features (Pehar *et al*., 2010). Importantly, haploinsufficiency of *MAPT* (the gene encoding tau) can rescue the synaptic deficits, suggesting that the hyperphosphorylation of tau is crucial for the synaptic-associated phenotype (Pehar *et al*., 2010).

Here, we report that p44 can bind to the promoter of several tau kinases and regulate their transcription, thus resulting in increased mRNA and protein levels. Although we limited our attention to Dyrk1A, GSK3β, and Cdk5/p35/p39, it is possible that additional kinases might be regulated in a similar fashion. Our results also indicate that p44 can regulate transcription of the above kinases, either alone or in concert with p53. Whether the interaction with the full-length protein adds a further level of biological complexity remains to be determined. In fact, some p53 targets are similarly regulated by p53 and p44 while others appear to be differently regulated (Courtois *et al*., 2002; Maier *et al*., 2004). The fact that the progeroid phenotype of p44^+/+^ mice depends on p53 (Maier *et al*., 2004) would suggest that the two proteins act in concert, at least *in vivo* and with age-associated events. p44 forms oligomers with p53 (Courtois *et al*., 2002; Ghosh *et al*., 2004; Powell *et al*., 2008). Data from different experimental settings also indicate that p44 can affect half-life, post-translational modifications, and promoter-affinity of the p53/p44 complex [reviewed in (Campisi, 2004; Scrable *et al*., 2005)]. Therefore, it is possible that changes in the expression levels of p44 can alter the biological properties of the p53/p44 transcriptional complex. This would explain why mice overexpressing p44 develop a progeroid phenotype (Maier *et al*., 2004) while mice overexpressing full-length p53 do not (Garcia-Cao *et al*., 2002).

During aging, a large segment of the population will experience some degree of cognitive decline [reviewed in (Pehar & Puglielli, 2012)]. Postmortem human studies have also shown that brain aging is accompanied by progressive accumulation of hyperphosphorylated tau in the form of intraneuronal inclusions (referred to as NFT). The severity of the tau alterations appears to increase inexorably throughout aging (Hebert *et al*., 2003; Thal *et al*., 2004). Similar alterations – although more severe – are observed in individuals affected by late-onset AD [reviewed in (Pehar & Puglielli, 2012)]. Importantly, aging is the most important risk factor for late-onset AD [reviewed in (Pehar & Puglielli, 2012)]. It is also worth stressing that several studies have analyzed mRNA changes that occur in the aging brain. These studies have been performed in mice, rats, nonhuman primates, and humans. They all show consistent changes that are limited to a small subset of genes (<5% of all genes expressed in the brain) (Lee *et al*., 2000; Jiang *et al*., 2001; Blalock *et al*., 2003; Lu *et al*., 2004; Fraser *et al*., 2005). Interestingly, the resulting ‘aging profile’ appears to overlap with the ‘AD profile’ suggesting a continuum between normal aging of the brain and AD [discussed in (Pehar & Puglielli, 2012)]. Obviously, normal aging and AD are very different and not every old individual will develop AD. However, it is still possible that similar molecular events might underline the cognitive decline associated with normal aging and AD.

Tau is a microtubule-binding protein that participates in the stabilization of microtubules. It can be phosphorylated at different sites, and the degree of phosphorylation inversely correlates with binding to microtubules. Abnormally (hyper) phosphorylated tau dissociates from microtubules and aggregates in NFT. *Mapt* mutations have been associated with hereditary forms of frontotemporal dementias; additionally, tau polymorphisms appear to act as genetic risk factors for sporadic progressive supranuclear palsy and corticobasal degeneration (Lee *et al*., 2001). Abnormal phosphorylation and aggregation of tau are also an essential pathological feature of AD. Finally, tau hyperphosphorylation has been linked to the memory deficits of different mouse models of human neurodegenerative diseases (Lee *et al*., 2001; Kurz & Perneczky, 2009).

The results reported here, together with those obtained with p44^+/+^ transgenic mice (Pehar *et al*., 2010), indicate that p44 might be involved in the increased phosphorylation and altered metabolism of tau that characterizes aging. Genetic disruption of the *TP53* gene did not affect the mRNA levels of tau kinases, indicating that neither p53 nor p44 are responsible for their baseline levels. However, overexpression of p44 alone (in cultured cells and in the mouse) was able to alter their expression profile and affect the phosphorylation status of tau. These results should be viewed together with the fact that p44 is upregulated in an age-dependent fashion in the mouse brain. Therefore, p44 might be involved – at least in part – with the abnormal phosphorylation of tau and the increased propensity to the cognitive decline that characterizes aging. The possible association with late-onset AD remains to be fully explored.

## Experimental procedures

### Cells, brain tissue, and animals

Cells used throughout this study were cultured as described before (Costantini *et al*., 2006; Jonas *et al*., 2010; Pehar *et al*., 2012a). For neuronal cultures, hippocampi and frontal cortices were dissected from embryonic-day 16–18 (E16–18) mice; cultures were established as described before (Costantini *et al*., 2005, 2006).

Intact frozen brains from young and old wild-type/nontransgenic mice (4, 18, and 32 months of age) were obtained from the National Institute on Aging (NIA) Rodent Tissue Bank. p44^+/+^ mice were described before (Maier *et al*., 2004; Costantini *et al*., 2006; Pehar *et al*., 2010); p75^NTR/ExonIII−/−^ (simply called p75^NTR−/−^ throughout this study) mice were obtained from The Jackson Laboratory (Bar Harbor, ME, USA) and were described before (Costantini *et al*., 2005); intact frozen brains from p75^NTR+/+^ mice were obtained from JSW Research (Graz, Austria); Igf1r^+/−^ mice were kindly provided by Dr. A. Efstratiadis (Columbia University, New York) and were described before (Pehar *et al*., 2010). Animal experiments were carried out in accordance with the NIH Guide for the Care and Use of Laboratory Animals and were approved by the Institutional Animal Care and Use Committee of the University of Wisconsin-Madison and the Madison Veterans Administration Hospital.

### Western blot analysis

Protein extracts were prepared as described before (Costantini *et al*., 2006; Ko & Puglielli, 2009; Pehar *et al*., 2012b) in GTIP (10 mm Tris, pH 7.6, 2 mm EDTA, 150 mm NaCl) buffer supplemented with 1% Triton X-100 (Roche Applied Science, Indianapolis, IN, USA), 0.25% Nonidet P-40 (Roche Applied Science), complete protein inhibitor mixture (Roche Applied Science), and phosphatase inhibitors (mixture set I and set II; Calbiochem, Billerica, MA, USA). Protein concentration was measured by the bicinchoninic acid method (Pierce, Rockford, IL, USA). Western blotting was performed on a 4–12% BisTris SDS–PAGE system (NuPAGE; Invitrogen, Grand Island, NY, USA) as described (Costantini *et al*., 2006; Ko & Puglielli, 2009; Pehar *et al*., 2012b). The following antibodies were used: antiphospho IGF1-R (monoclonal; Cell Signaling), antiphospho-Akt (polyclonal; Cell Signaling), antitotal tau (monoclonal-T46; Zymed/Invitrogen), antiphosho-tau (monoclonal-AT8; Thermo Scientific, Fitchburg, WI, USA), anti-Dyrk1a (polyclonal; Cell Signaling), anti-GSK3β total (monoclonal; Cell Signaling), anti-GSK3β S9 (monoclonal; Cell Signaling), anti-GSK3β Y279/Y216 (monoclonal; Abcam, Cambridge, MA, USA), anti-Cdk5 (polyclonal; Cell Signaling), anti-p35/p25 (monoclonal; Cell Signaling), anti-p39 (polyclonal; Cell Signaling), antitubulin (monoclonal; Covance), anti-H3 (polyclonal, EMD Millipore, Billerica, MA, USA), anti-GAPDH (monoclonal; Abcam), anti-actin (polyclonal; Cell Signaling), anti-p53 (monoclonal-PAb240; Ancell, Bayport, MN, USA); anti-p53 (monoclonal; EMD Millipore), anti-N-BACE1 (monoclonal; R&D Systems, Minneapolis, MN, USA), anti-SREBP-2 (polyclonal; BD-Biosciences, San Jose, CA, USA). Samples were imaged as described (Costantini *et al*., 2006; Ko & Puglielli, 2009; Pehar *et al*., 2012b).

For densitometric analysis, we used the NIH image program and the LI-COR Odyssey imaging system (Labworks Image Acquisition and Analysis Software 4.5; Lincoln, NE, USA). For partially overlapping bands, we also used pixel density (for signal area) quantification with Adobe Photoshop.

### Real-time PCR

Real-time PCR was performed as described before (Jonas *et al*., 2010; Pehar *et al*., 2012a). The cycling parameters were as follows: 95 °C, 10 s; 55 or 59 °C, 10 s; 72 °C, 15 s. Controls without reverse transcription were included in each assay. Specific primers are shown in Supplemental Table S1. Gene expression levels were normalized against GAPDH levels and expressed as the percentage of control.

### Chromatin immunoprecipitation (ChIP) analysis

ChIP analysis was performed as described before (Ko & Puglielli, 2007). Briefly, human neuroblastoma (SH-SY5Y) cells were cultured in complete medium in 150-mm Petri dishes until 70–80% confluent. Cells were then fixed by adding 280 μL of 37% formaldehyde (Sigma, St. Louis, MO, USA) to the 10 mL of culture medium for 10 min at 37 °C, harvested, and processed for ChIP using a commercially available kit (EMD Millipore). p53:p44-DNA immune complexes were precipitated with indicated antibodies. PCR was carried out using primer sets listed in Supplemental Table S2.

### Biotin–streptavidin pull down assay

The biotin–streptavidin pull down assay was performed as described before (Ko & Puglielli, 2007). Briefly, 1 μg of biotin double-stranded DNA was used as probe. Individual probes corresponded to the fragments amplified by the primers sets used for ChIP (see Supplemental Table S2). Each probe was incubated with nuclear extracts of MEF-p44^+/+^ and Saos2 cells for 30 min at room temperature. Incubation, pull-down, and successive analysis was conducted as described (Ko & Puglielli, 2007). For Western blot, samples were separated on a 4–12% Bis-Tris gel system (NuPAGE; Invitrogen) and probed with antibody PAb240 against the DNA-binding motif of p53/p44.

### Luciferase reporter assay

The ChIP-positive promoter fragments were cloned into a promoterless pGL3 plasmid (Promega, Madison, WI, USA). Human osteosarcoma (Saos2) cells, which are null for p53 (Masuda *et al*., 1987), were transfected with 1 μg of the promoter–reporter construct as well as the empty vector along with 0.1 μg of *Renilla* luciferase (Promega). Firefly and *Renilla* luciferase activities were measured 36 h after transfection with a dual luciferase kit (Promega) and expressed as relative luciferase activity. Cotransfected *Renilla* luciferase was used to normalize for transfection efficiency.

### Statistical analysis

Data analysis was performed using GraphPad InStat 3.06 statistical software (GraphPad Software, San Diego, CA, USA). Data are expressed as mean ± SD and were analyzed using Student’s *t-*test or one-way analysis of variance followed by Tukey–Kramer multiple comparisons test. Differences were declared statistically significant if *P* ≤ 0.05.

## Supporting Information

**Table S1** Primers used for real-time PCR.

**Table S2** Primers used for ChIP.

## References

[b1] Azevedo FA, Carvalho LR, Grinberg LT, Farfel JM, Ferretti RE, Leite RE, Jacob Filho W, Lent R, Herculano-Houzel S (2009). Equal numbers of neuronal and nonneuronal cells make the human brain an isometrically scaled-up primate brain. J. Comp. Neurol.

[b2] Ballatore C, Lee VM, Trojanowski JQ (2007). Tau-mediated neurodegeneration in Alzheimer’s disease and related disorders. Nat. Rev. Neurosci.

[b3] Bauer JH, Helfand SL (2006). New tricks of an old molecule: lifespan regulation by p53. Aging Cell.

[b4] Blalock EM, Chen KC, Sharrow K, Herman JP, Porter NM, Foster TC, Landfield PW (2003). Gene microarrays in hippocampal aging: statistical profiling identifies novel processes correlated with cognitive impairment. J. Neurosci.

[b5] Bourdon JC (2007). p53 and its isoforms in cancer. Br. J. Cancer.

[b6] Bourdon JC, Fernandes K, Murray-Zmijewski F, Liu G, Diot A, Xirodimas DP, Saville MK, Lane DP (2005). p53 isoforms can regulate p53 transcriptional activity. Genes Dev.

[b7] Campisi J (2004). Fragile fugue: p53 in aging, cancer and IGF signaling. Nat. Med.

[b8] Costantini C, Weindruch R, Della Valle G, Puglielli L (2005). A TrkA-to-p75NTR molecular switch activates amyloid beta-peptide generation during aging. Biochem. J.

[b9] Costantini C, Scrable H, Puglielli L (2006). An aging pathway controls the TrkA to p75(NTR) receptor switch and amyloid beta-peptide generation. EMBO J.

[b10] Courtois S, Verhaegh G, North S, Luciani MG, Lassus P, Hibner U, Oren M, Hainaut P (2002). DeltaN-p53, a natural isoform of p53 lacking the first transactivation domain, counteracts growth suppression by wild-type p53. Oncogene.

[b11] Ferrer I, Barrachina M, Puig B, Martinez de Lagran M, Marti E, Avila J, Dierssen M (2005). Constitutive Dyrk1A is abnormally expressed in Alzheimer disease, Down syndrome, Pick disease, and related transgenic models. Neurobiol. Dis.

[b12] Fraser HB, Khaitovich P, Plotkin JB, Paabo S, Eisen MB (2005). Aging and gene expression in the primate brain. PLoS Biol.

[b13] Garcia-Cao I, Garcia-Cao M, Martin-Caballero J, Criado LM, Klatt P, Flores JM, Weill JC, Blasco MA, Serrano M (2002). “Super p53” mice exhibit enhanced DNA damage response, are tumor resistant and age normally. EMBO J.

[b14] Ghosh A, Stewart D, Matlashewski G (2004). Regulation of human p53 activity and cell localization by alternative splicing. Mol. Cell. Biol.

[b15] Goldstein JL, DeBose-Boyd RA, Brown MS (2006). Protein sensors for membrane sterols. Cell.

[b16] Hebert LE, Scherr PA, Bienias JL, Bennett DA, Evans DA (2003). Alzheimer disease in the US population: prevalence estimates using the 2000 census. Arch. Neurol.

[b17] Hooper C, Killick R, Lovestone S (2008). The GSK3 hypothesis of Alzheimer’s disease. J. Neurochem.

[b18] Jiang CH, Tsien JZ, Schultz PG, Hu Y (2001). The effects of aging on gene expression in the hypothalamus and cortex of mice. Proc. Natl Acad. Sci. USA.

[b19] Jonas MC, Pehar M, Puglielli L (2010). AT-1 is the ER membrane acetyl-CoA transporter and is essential for cell viability. J. Cell Sci.

[b20] Ko MH, Puglielli L (2007). The sterol carrier protein SCP-x/pro-SCP-2 gene has transcriptional activity and regulates the Alzheimer disease gamma-secretase. J. Biol. Chem.

[b21] Ko MH, Puglielli L (2009). Two Endoplasmic Reticulum (ER)/ER Golgi Intermediate Compartment-based Lysine Acetyltransferases Post-translationally Regulate BACE1 Levels. J. Biol. Chem.

[b22] Kurz A, Perneczky R (2009). Neurobiology of cognitive disorders. Curr. Opin. Psychiatry.

[b23] Lee CK, Weindruch R, Prolla TA (2000). Gene-expression profile of the ageing brain in mice. Nat. Genet.

[b24] Lee VM, Goedert M, Trojanowski JQ (2001). Neurodegenerative tauopathies. Annu. Rev. Neurosci.

[b25] Lu T, Pan Y, Kao SY, Li C, Kohane I, Chan J, Yankner BA (2004). Gene regulation and DNA damage in the ageing human brain. Nature.

[b26] Maier B, Gluba W, Bernier B, Turner T, Mohammad K, Guise T, Sutherland A, Thorner M, Scrable H (2004). Modulation of mammalian life span by the short isoform of p53. Genes Dev.

[b27] Marcel V, Perrier S, Aoubala M, Ageorges S, Groves MJ, Diot A, Fernandes K, Tauro S, Bourdon JC (2010). Delta160p53 is a novel N-terminal p53 isoform encoded by Delta133p53 transcript. FEBS Lett.

[b28] Masuda H, Miller C, Koeffler HP, Battifora H, Cline MJ (1987). Rearrangement of the p53 gene in human osteogenic sarcomas. Proc. Natl Acad. Sci. USA.

[b29] Ohki R, Kawase T, Ohta T, Ichikawa H, Taya Y (2007). Dissecting functional roles of p53 N-terminal transactivation domains by microarray expression analysis. Cancer Sci.

[b30] Patrick GN, Zukerberg L, Nikolic M, de la Monte S, Dikkes P, Tsai LH (1999). Conversion of p35 to p25 deregulates Cdk5 activity and promotes neurodegeneration. Nature.

[b31] Pehar M, Puglielli L, Perloft JW, Wong AH (2012). Molecular and cellular mechanisms linking aging to cognitive decline and Alzheimer’s disease. Cell Aging.

[b32] Pehar M, O’Riordan KJ, Burns-Cusato M, Andrzejewski ME, del Alcazar CG, Burger C, Scrable H, Puglielli L (2010). Altered longevity-assurance activity of p53:p44 in the mouse causes memory loss, neurodegeneration and premature death. Aging Cell.

[b33] Pehar M, Jonas MC, Hare TM, Puglielli L (2012a). SLC33A1/AT-1 Protein Regulates the Induction of Autophagy Downstream of IRE1/XBP1 Pathway. J. Biol. Chem.

[b34] Pehar M, Lehnus M, Karst A, Puglielli L (2012b). Proteomic assessment shows that many endoplasmic reticulum (ER)-resident proteins are targeted by N{epsilon}-lysine acetylation in the lumen of the organelle and predicts broad biological impact. J. Biol. Chem.

[b35] Powell DJ, Hrstka R, Candeias M, Bourougaa K, Vojtesek B, Fahraeus R (2008). Stress-dependent changes in the properties of p53 complexes by the alternative translation product p53/47. Cell Cycle.

[b36] Rodier F, Campisi J, Bhaumik D (2007). Two faces of p53: aging and tumor suppression. Nucleic Acids Res.

[b37] Ryoo SR, Jeong HK, Radnaabazar C, Yoo JJ, Cho HJ, Lee HW, Kim IS, Cheon YH, Ahn YS, Chung SH, Song WJ (2007). DYRK1A-mediated hyperphosphorylation of Tau. A functional link between Down syndrome and Alzheimer disease. J. Biol. Chem.

[b38] Saez ET, Pehar M, Vargas MR, Barbeito L, Maccioni RB (2006). Production of nerve growth factor by beta-amyloid-stimulated astrocytes induces p75NTR-dependent tau hyperphosphorylation in cultured hippocampal neurons. J. Neurosci. Res.

[b39] Schmid G, Strosznajder JB, Wesierska-Gadek J (2006). Interplay between the p53 tumor suppressor protein family and Cdk5: novel therapeutic approaches for the treatment of neurodegenerative diseases using selective Cdk inhibitors. Mol. Neurobiol.

[b40] Scrable H, Sasaki T, Maier B (2005). DeltaNp53 or p44: priming the p53 pump. Int. J. Biochem. Cell Biol.

[b41] Thal DR, Del Tredici K, Braak H (2004). Neurodegeneration in normal brain aging and disease. Sci. Aging Knowledge Environ.

[b42] Ungewitter E, Scrable H (2008). Antagonistic pleiotropy and p53. Mech. Ageing Dev.

