# Supplementary material for: P44, the ‘longevity-assurance’ isoform of P53, regulates tau phosphorylation and is activated in an age-dependent fashion
Source: Aging Cell. 2014 Feb 25;13(3):449–56. doi: 10.1111/acel.12192 (PMC4032616; doi:10.1111/acel.12192)
Supplement: Supplementary file 1 — Table S1 Primers used for real-time PCR. [file acel0013-0449-sd1.pdf]

Supplemental Table S1. Primers used for real-time PCR.

Primer pair	Sequence
**CDK5**	Forward: 5’-AAGCGTGTCAGGCTGGATGATG-3’
	Reverse: 5’-ACGTTGCGGCTGTGACAGAATC-3’
**CDK5P35**	Forward: 5’-ATGCCGACCCACACTATTTCACAC-3’
	Reverse: 5’-TCCTCCTGACCGCTCTCATTCTTC -3’
**CDK5P39**	Forward: 5’-GGCCGTCCGTGCTCATCTCGGCGCTCA -3’
	Reverse: 5’-CGGCCCTTGCGGAGAAGGTTCTCGCGGTTGCG -3’
**DYRK1A**	Forward: 5’-TTGGCAGCTCTTGTCAGTTGGG-3’
	Reverse: 5’-TTCTTGCTTTCGGTGCTTGGTC-3’
**GSK3β**	Forward: 5’-CACTCAAGAACTGTCAAGTAAC-3’
	Reverse: 5’-CATTAGTATCTGAGGCTGCTG-3’
**MAPT**	Forward: 5’-CCCCCTAAGTCACCATCAGCTAGT-3’
	Reverse: 5’-CACTTTGCTCAGGTCCACCGGC-3’
**GAPDH**	Forward: 5’-AAGATGGTGATGGGCTTCCCG-3’
	Reverse: 5’-TGGCAAAGTGGAGATTGTTGCC-3’
