# Supplementary material for: P44, the ‘longevity-assurance’ isoform of P53, regulates tau phosphorylation and is activated in an age-dependent fashion
Source: Aging Cell. 2014 Feb 25;13(3):449–56. doi: 10.1111/acel.12192 (PMC4032616; doi:10.1111/acel.12192)
Supplement: Supplementary file 2 — Table S2 Primers used for ChIP. [file acel0013-0449-sd2.pdf]

Supplemental Table S2. Primers used for ChIP.

Primer pair	Sequence	A. Temp.	Prod. Size
**CDK5**	Forward: 5’-TTGTCCTCGGTCTCGGGCATTGCG-3’	60.3 ^{o}C	237 bp
	Reverse: 5’-GCAGGAACATCTCGAGATTCCATT -3’		
**CDK5P35**	Forward: 5’-GGGTTGCGCGGGCGCCGAGGA-3’	62 ^{o}C	213 bp
	Reverse: 5’-GCGCACGGTGCGGGTAGGACG-3’		
**CDK5P39**	Forward: 5’-AGCCACCACCCCTTCTCAACTCTG-3’	62 ^{o}C	207 bp
	Reverse: 5’-GCCTCACGCGGCCAATCCGCTCCC-3’		
**DYRK1A**	Forward: 5’-TTTGGGATTGTAGTTATGTTAGAT-3’	58 ^{o}C	191 bp
	Reverse: 5’-CAAAACTTGAGTCACCTGTATGCATCG-3’		
**GSK3β**	Forward: 5’-CCGAGGAAAATATAATATTCGA-3’	55.3 ^{o}C	250 bp
	Reverse: 5’-CCTCTTGGCTTTTCACTCCTTTTG-3’		

A. Temp., annealing temperature; *Prod. Size*, product size.
